# Solving problems that do not exist and not solving problems that do exist, magnetic personalities included

**DOI:** 10.3325/cmj.2021.62.640

**Published:** 2021-12

**Authors:** Charles H. Calisher

**Affiliations:** calisher@cybersafe.net

“Politicians are the same all over: they promise to build a bridge even where there is no river.” – Nikita Khrushchev

If everyone were to be usefully vaccinated against severe acute respiratory virus type 2 (SARS-CoV-2), the causative agent of pandemic COVID-19, and each person were to mount an adequate immunological response, the number of cases of this disease would decrease to none or very few (some people inevitably will not respond as expected), unless the virus mutates even further. Because people do not all respond to public health and other governmental suggestions, this will not happen. Not everyone will get vaccine-immunized, either because of lack of access to one of the vaccines, because none of these vaccines is 100% effective, or because some people will refuse the vaccine, including those who pray to God to spare them, the same God who invented this virus in the first place. Even if vaccinated, immunologic responses to one or more of the available vaccines vary from typical to atypical, compounding the effect of asymptomatic infections on transmission of the virus; individuals may become infected and transmit the virus to recipients who are not immune – and the disaster continues. Even at this relatively early stage, the need for annual booster doses is being suggested.

There have arisen many nonsensical comments, conjectures, and suggestions regarding the constituents of the new coronavirus vaccines. I suppose these have been intended to confuse or frighten people who have hesitated to be vaccinated, perhaps even convincing them to not be vaccinated, or to further agitate people who have been vaccinated. Among the nonsense that has circulated has been the possibility that the vaccines make the vaccinee magnetic. The US Centers for Disease Control and Prevention has found it necessary to suppress this rubbish and has stated: “Receiving a COVID-19 vaccine will not make you magnetic, including at the site of vaccination, which is usually your arm. COVID-19 vaccines do not contain ingredients that can produce an electromagnetic field at the site of your injection. All COVID-19 vaccines are free from metals such as iron, nickel, cobalt, lithium, and rare earth alloys, as well as any manufactured products such as microelectronics, electrodes, carbon nanotubes, and nanowire semiconductors. In addition, the typical dose for a COVID-19 vaccine is less than a milliliter, which is not enough to allow magnets to be attracted to your vaccination site even if the vaccine was filled with a magnetic metal.” (1). Laughingly, an anti-vaccine nurse [Note: not all nurses are of equal intelligence and training] in Ohio recently testified to a US political party’s hearing that having been vaccinated made her body magnetic. Of course, she failed when she tried to demonstrate the effect. If she is not married, that is a little more evidence that she is not magnetic. More recently, a report circulated in South Korea that Covid-19 vaccines contain electronic devices that can activate light bulbs. This is neither true nor enlightening. An Urdu-language newspaper in Pakistan warned that “[menstrual] Periods of 4000 British women have stopped, their biological systems have been messed up”. It reported that, although nearly 4000 women reported period problems after receiving a Covid-19 vaccine, health authorities found no causal link (2). Even infertility has been suggested as a result of administrations of these vaccines. Stupidity is not a result of vaccination; it is a cause of anti-vaccination efforts, usual a political cause.

The latest US ex-President suggested that the use of hydroxychloroquine is beneficial and that injecting chlorine products or other disinfectants was another possible cure for infection with SARS-CoV-2 (after he said that, disinfectant poisonings increased as much as 121% the next month). Indeed, the US Food and Drug Administration removed its emergency use authorization after studies showed a risk associated with use of hydroxychloroquine. More recently, ivermectin, a macrocyclic lactone used to successfully treat worm infections, mostly of veterinary patients, has been tried informally as an anti-viral, although its use has been considered by many as “dangerous.” This drug has now been associated with many human poisonings, with cases being reported to governmental poisoning centers. Listening to ignorant people is not a safe or useful replacement for paying attention to those who are at the very least “enlightened.” I wonder what happened to all those dollars spent to educate the American public.

Other anti-vaccination people have said that COVID-19 vaccines make people produce a spike protein that is a toxin and can spread to other parts of the body and damage organs and that coronavirus cases without symptoms are not real, that an asymptomatic patient is simply a healthy person. From my own careful scientific survey of non-vaccinees, two of three (66.6667%) people admitted being afraid of a needle-stick.

Recently I read that Stephen Hawking, the famous theoretical physicist, suggested that “With climate change, overdue asteroid strikes, epidemics and population growth, our own planet is increasingly precarious.” In addition, we have many world leaders with control over nuclear weapons and we have to depend on their mental stability to not use them. Plus, an extremely large 2000-square-mile piece of ice (about the size of Trinidad and Tobago and twice the size of Luxembourg) is about to break off the Antarctic Peninsula and sea levels might rise because the ice shelf was keeping ice from nearby glaciers from sliding into the water. So, between the threat of climate change, nuclear weapons, gigantic icebergs melting in the sea lanes, uncontrollable asteroids, pandemics, and overpopulation, not to speak of the nearly 900 000 000 people who have been vaccinated against SARS-CoV-2 and are allegedly magnetized, we are in sad condition as a world. No wonder the Powers That Be are making preliminary efforts to see what is and who are on Mars and how habitable it might be. I am not concerned about overpopulation; that situation will likely take care of itself, although we might have to replace meat with seaweed or grass, which my bovid friends tell me is delicious.

What does concern me are the nuclear weapons. It might be better if we shipped those weapons and their possessors-handlers to Mars and let the Martians take care of things. That is, if there is life on Mars; whatever its form, I would not look forward to sharing Earth with them, antibiotics likely would not be sufficient or efficacious. Introducing that magnetized woman in Ohio to Little Green People might be an interesting solution.

The magnetism story intrigues me, however. What if the Ohio nurse (who also suggested that the magnetism the vaccines causes is manipulated by 5G telecommunications towers) and the osteopathic medical doctor who agrees with her are right, even though their attempted demonstration of such magnetism failed miserably? I would be in favor of seeing a positive test. I personally have no magnetism, as can be attested by the many girls who refused to date me when I was a teenager, but I would like some (magnetism). If I was magnetic, I could hire myself out to mining companies, automobile dealers, people who have lost coins in their sofas, those searching for pirates’ treasures in the Caribbean or for coins on beaches, as well as help my wife find my eyeglasses. On the other hand, she also has been vaccinated, so that might be counterproductive.

Most people I know work hard to see to it that their children absorb as much information as they possibly can, that they get an education at the highest level they can tolerate. After that is accomplished they forget or ignore why they did all that and complain about “intellectuals” who have, over the years, provided antibiotics, developed robotic surgery, detected and identified pathogen genomes by polymerase chain reaction assays, improved treatments of infectious diseases, facilitated repair of injured nerves, muscles, ligaments and tendons, developed lens replacement surgery to remove the natural lens from eyes that have developed opacifications (cataracts), established methods for use in dental implants, decreased the weights of automobiles while, at the same time, making them safer, and brought cell phones to most of the world. None of these advances was made by people making good guesses or responding to their “feelings”.

What if COVID-19 was plague or smallpox? Would governments make antibiotic treatment or vaccination voluntary? Would some proportion of people consider these hoaxes (while bodies of victims lie in the streets)? Would at least some people refuse to allow their children to be administered curatives or preventatives? It already is compulsory in some countries to be vaccinated against a variety of viruses before being allowed to attend public schools or to marry, and it is even required to wear seatbelts in vehicles in some countries, so why not be vaccinated against this coronavirus?

There certainly is some unknown risk to being administered a vaccine that had been spoken of as being “experimental” or even “a new approach”? However, the risk of acquiring an infection with SAR-CoV-2 is much greater if one does not become vaccinated. As important, lack of 100% population immunity will continue to provide an environment in which evolutionary variants of SARS-CoV-2, such as the delta and omicron variants, will continue to be generated. Imagine how many variants might have been generated by going through many trillions of lung cells in China, India, and elsewhere. Politicians and others who argue and work against vaccination programs and vaccines might easily be considered conspirators against the good health of their own fellow citizens. Why would decent people want to do that? The sooner we get rid of or at least minimize the occurrence of this horrible virus, the better. The world has other problems, large problems, problems with no current solutions, and will have more in the future. Not being vaccinated against this pandemic coronavirus is not only anti-social, it indicates a greater love for political hypotheses than love for one’s own children and those of others.
